# ^18^F-FDG PET/CT imaging in the diagnosis of drug-induced lung disease and pulmonary infection in lymphoma

**DOI:** 10.1097/MD.0000000000027107

**Published:** 2021-09-17

**Authors:** Tingting Lu, Guoren Yang

**Affiliations:** a Medical Imaging Center, Xi’An QinHuang Hospital, Xi’an, Shanxi, China; b Department of Nuclear Medicine, Shandong Cancer Hospital and Institute, Shandong First Medical University and Shandong Academy of Medical Sciences, Jinan, China.

**Keywords:** ^18^Fluorodeoxyglucose (^18^F-FDG), chemotherapy (CHT), drug-induced interstitial lung disease (DILD), lymphoma, maximum standardized uptake value (SUV_max_), positron emission tomography/computed tomography (PET/CT), pulmonary infection (PI)

## Abstract

**Objective:**

Lymphoma is a hematological disease with high prevalence. Multi-cycle chemotherapy (CHT) or local radiotherapy is applied usually; however, adverse events have been reported, such as drug-induced lung disease (DILD). Positron emission tomography/computed tomography (PET/CT) is often used to evaluate the lesion, treatment effect, and prognosis of lymphoma. We investigated DILD and pulmonary infection (PI) after multi-cycle CHT in lymphoma patients, to identify DILD and PI, provide guidance for later treatment for them.

**Methods:**

In all, 677 patients diagnosed with lymphoma and who underwent CHT were included. These patients underwent ^18^fluorodeoxyglucose (^18^F-FDG) PET/CT before and after CHT at Shandong Cancer Hospital (affiliated with Shandong University) between April 2015 and November 2019. Fifty patients developed DILD, 41 patients had lung infections; lesion characteristics were analyzed based on clinical characteristics, laboratory examinations, and PET/CT imaging.

**Results:**

Among the 677 lymphoma patients, there were 50 cases of DILD, with an incidence rate of 7.4%. PET/CT showed an elevated ^18^fluorodeoxyglucose uptake lung background, septal thickening and reticulation, multiple ground glass-like shadows, and grid-shaped blur shadows, which were more common in the lung periphery and under the pleura. The maximum standardized uptake value in the lung was 2.45 ± 0.52. Pulmonary infections occurred in 41 patients, and the maximum standardized uptake value was 4.05 ± 1.42. Age, sex, CHT cycle, Ann-Arbor stage, and lymphocyte levels were not significantly different between DILD and PI patients. Leukocyte and neutrophils showed significant differences; the PI patients had increased laboratory indexes of leukocyte and neutrophils. The mean number of CHT cycles was 4 cycles for DILD and PI.

**Conclusions:**

PET/CT imaging has high sensitivity and detection rates for primary and metastatic lymphoma lesions. DILD mostly occurs in the middle and late stages of CHT. Laboratory tests and PET/CT can evaluate the lesions and treatment effects, and provide guidance for subsequent treatment plans for patients.

## 1 Introduction

Lymphoma is a hematological malignant tumor formed by the abnormal cloning of lymphocytes and precursor cells. With decreased human immunity, drug resistance, and the long-term use of immunosuppressive drugs in patients, the prevalence of lymphoma has significantly increased.^[1]^ Lymphomas are divided into Hodgkin lymphoma (HL) and non-Hodgkin lymphoma (NHL), and they comprise diverse subtypes.^[2,3]^ Since the 1940s, lymphoma has mainly been treated with multi-cycle chemotherapy (CHT), with a high cure rate and low recurrence rates. Due to medical advances and research into individual differences, the treatment for lymphoma has become more precise, with the main CHT regimens including the combination of rituximab, cyclophosphamide, doxorubicin, vincristine, and prednisone (R-CHOP), pegylated liposomal doxorubicin instead of doxorubicin in combination with rituximab, cyclophosphamide, vincristine, and prednisone (R-CDOP), and doxorubicin (**A**driamycin), **b**leomycin, **v**inblastine, and **d**acarbazine (ABVD). However, a series of adverse events have been reported, including hematological toxicities, bone marrow suppression, drug-induced lung disease (DILD), cardiologic complications, and liver injury. DILD is the most frequent complication, refers to the occurrence of new lesions while the primary lesions shrink or disappear during CHT, and there is no significant correlation with the primary lesions. The incidence of DILD is 5.5% to 10%^[4]^ and disappears following drug withdrawal or steroid usage generally.

DILD can be monitored through clinical symptoms and medical imaging findings. Most patients have no obvious clinical symptoms during the early stages,^[5]^ but a few patients may have symptoms such as a cough, fever, and dyspnea. Imaging methods include chest radiographs, computed tomography (CT), mainly high-resolution CT (HRCT), and positron emission tomography/CT (PET/CT) scanning. Chest radiography show thickening and disordered lung texture, alveolar and interstitial infiltrates, and even bilateral consolidation; however, it does not show specific manifestations. CT shows septal thickening and reticulation, multiple ground glass-like shadow or grid-shaped blur shadow, and lung consolidation,^[6,7]^ which is common in lung periphery and under the pleura but cannot be used distinguish among DILD, pulmonary infection (PI), and lung infiltration. PET/CT can be used not only for diagnosis and staging but also to assess the treatment and related adverse by combining anatomical and functional images.^[8,9]^ Few studies have evaluated DILD and PI in lymphoma patients using PET/CT.^[10]^ In this study, we retrospectively reviewed lymphoma patients’ clinical characteristics, laboratory examinations, and PET/CT imaging to investigate the prevalence of DILD and PI, evaluate the therapeutic effects, and guide future treatment.

## 2 Materials and methods

### 2.1 General information

A total of 677 patients who were diagnosed with lymphoma and underwent CHT were enrolled. These patients also underwent ^18^fluorodeoxyglucose (^18^F-FDG) PET/CT before, between, and after their CHT at Shandong Cancer Hospital (affiliated with Shandong University), between April 2015 and November 2019. The lymphoma patients were retrospectively reviewed, and eligible patients were included. There were 124 patients with HL and 553 patients with NHL. Of these, 50 were found to have DILD, and this included 19 males and 31 females, with an average age of 53.2 ± 17.3 (median 55.5). Forty-one patients had PI, and this included 24 males and 17 females, with an average age of 56.1 ± 18.8 (median 56.0). The clinical characteristics, laboratory examinations, and PET/CT images were analyzed. The criteria for a diagnosis of DILD were as follows:^[4,11]^ 1) history of lymphoma treatment and no other disease that affected lung function; 2) normal lung function before CHT and imaging characteristics that could not be explained by infection during CHT; 3) clinical symptoms that could not be explained by infection, such as fever, cough, and dyspnea; 4) no pathogenic bacteria present in sputum cultures; 5) PET/CT scans showing an increased ^18^F-FDG uptake background, multiple ground glass-like shadows, or grid-shaped blur shadows, or even lung consolidation; and 6) imaging and clinical symptoms disappeared following drug withdrawal and corticosteroid usage. The PI criteria were as follows: 1) obvious clinical symptoms such as cough, fever, and sputum; 2) abnormal laboratory results (white blood cells, neutrophil counts, and sputum cultures); 3) scattered or local patchy shadows or consolidation in PET/CT images; and 4) symptoms improved after anti-inflammatory treatment.

### 2.2 Instruments and methods

A total of 677 patients were examined using a Philips GEMINI TF Big Bore PET/CT. ^18^F-FDG was produced by a cyclotron in the nuclear department, and its radiochemical purity was greater than 95%. After patients had fasted for more than 6 h, 3.7 MBq/kg (0.1 mCi/kg) ^18^F-FDG was injected into a vein. The patients were asked to drink water (1500–2000 mL), and imaging commenced after 1 hour. The CT scans were taken from the cranial crest to the upper femur, at 120 kVp and 100 mAs, with a 512 × 512 matrix, a pitch of 0.75, and a layer thickness of 4.25 mm. PET images were collected with the same layer thickness and a 128 × 128 matrix every 2 min per bed. Next, with the patient maintaining his/her position, a deep inspiratory HRCT scan was performed using 64 × 1.25 mm detectors. PET images were reconstructed and fused using a Philips station.

### 2.3 Image analyses

Two experienced PET/CT physicians independently reviewed the treatment, lung lesion range, and morphology. They also identified DILD and PI, outlined the maximum standardized uptake value (SUV_max_), measured the mean standardized uptake value (SUV_mean_) in the ascending aortic cavity as the mediastinal blood pool background, calculated the SUV_max_/SUV_mean_ (standardized uptake ratio-blood pool, SUR-BP), analyzed ^18^F-FDG uptake and the clinical manifestations of DILD and PI patients, and guided future clinical treatments.

### 2.4 Statistical analyses

SPSS 25.0 was used for all statistical analyses. For measurement data, an independent samples *t* test was used for normally distributed data and a Mann–Whitney *U* test was used for non-normal distributions; the Chi-square test was used for count data, and a *P* value <.05 was taken to indicate statistical significance in two-sided tests.

## 3 Results

In 677 lymphoma patients, 50 developed DILD, with an incidence rate of 7.4%; 45 had NHL and 5 had HL. The standard CHT regimen was R-CHOP (29 cases), with the remainder being as follows (and see Table 1). Patients with DILD had 3 to 8 CHT cycles (mean: 4 cycles), of which 6 patients had fever and cough, and the remainder had no clinical symptoms. Pulmonary infections occurred in 41 patients, with an incidence rate of 6.1%; 34 had NHL and 7 had HL. The CHT regimens were as follows (see Table 1). In all, 29 patients had obvious symptoms such as fever, cough, and sputum; age (*P* = .722), sex (χ^2^ = 3.512, *P* = .060), CHT cycle (*P* = .200), Ann-Arbor stage (*P* = .192), and lymphocyte (*P* = .783) data showed no significant differences in the DILD and PI patients. Levels of leukocyte (*P* = .003) and neutrophils (*P* = .001) showed significant differences, with infected patients having increased laboratory indexes in terms of their leukocyte and neutrophils.

**Table 1:** Analysis of relevant data of patients with lymphoma DILD and PI.

		PET/CT lesion type		
		DILD	PI	*P* value
Sex	Male	19	24	.060
	Female	31	17	
Age (yrs)	53.2 ± 17.3	56.1 ± 18.8	0.722	
Average chemotherapy cycle	4	4	0.200	
Pathology	HL	5	7	.247
	NHL	45	34	
Treatment	R-CHOP	29	20	.273
	R-CDOP	7	2	
	CHOP	2	7	
	(Hyper-)ABVD	5	6	
	CVAD	4	1	
	Others	3	5	
Ann-Arb staging	1	9	3	.213
	2	14	12	
	3	13	11	
	4	14	15	
Before chemotherapy PET/CT	Lung SUV_max_	0.95 ± 0.21	0.89 ± 0.22	.156
	Mediastinum SUV_mean_	2.19 ± 0.43	2.20 ± 0.42	.595
	SUR/BP	0.45 ± 0.11	0.42 ± 0.09	.453
After chemotherapy PET/CT	Lung SUV_max_	2.45 ± 0.52	4.05 ± 1.42	<.001
	Mediastinum SUV_mean_	2.2 ± 0.42	2.07 ± 0.32	.158
	SUR/BP	1.15 ± 0.31	1.95 ± 0.87	.001
Laboratory examination	Leukocyte (×10^9^/L)	5.18 ± 2.28	7.84 ± 4.28	.003
	Neutrophils (×10^9^/L)	5.00 ± 10.59	12.5 ± 39.99	.001
	Lymphocytes (×10^9^/L)	1.09 ± 0.54	1.20 ± 1.23	.783

ABVD = doxorubicin (adriamycin), bleomycin, vinblastine, and dacarbazine, CDOP = pegylated liposomal doxorubicin, cyclophosphamide, vincristine, and prednisone, CVAD = cyclophosphamide, vincristine, adriamycin, and dexamethasone, DILD = drug-induced lung disease, HL = Hodgkin lymphoma, NHL = non-Hodgkin lymphoma, PET/CT = positron emission tomography/computed tomography, PI = pulmonary infection, R-CDOP = rituximab, pegylated liposomal doxorubicin, cyclophosphamide, vincristine, and prednisone, R-CHOP = rituximab, cyclophosphamide, doxorubicin, vincristine, and prednisone, SUR-BP = standardized uptake ratio-blood pool, *SUV*_*max*_ = The maximum standardized uptake value, SUV_mean_ = mean standardized uptake value.

Fifty patients developed DILD, and the PET/CT showed an elevated ^18^F-FDG uptake lung background, septal thickening, and reticulation, multiple ground glass-like shadows, and grid-shaped blur shadows,^[6,7]^ which were more common in the lung periphery and under the pleura. Eight patients had obvious lung consolidation, and 3 patients had an allergic lung injury, showing a grid-shaped thickening and mosaic-like appearance. Four patients had pleural effusion, and 1 had drug-induced liver injury. The lung SUV_max_ of the DILD patients before CHT was 0.5 to 1.3 (average 0.95), and the SUR-BP was 0.29 to 0.80 (average 0.45); the lung SUV_max_ after CHT was 1.40 to 3.50 (average 2.4), and the SUR-BP was 0.58 to 1.94 (average 1.15). Forty-one patients had PI, with a lung SUV_max_ before CHT of 0.50 to 1.30 (average 0.86), and a SUR-BP of 0.28 to 0.71 (average 0.42); the lung SUV_max_ after CHT was 2.10 to 9.90 (average 4.1) and the SUR-BP was 0.48 to 4.95 (average 1.94). Lung SUV_max_ (*P* = .156), mediastinal SUV_mean_ (*P* = .595) and SUR-BP (*P* = .453) before CHT, and mediastinal SUV_mean_ (*P* = .158) after CHT, were not significantly different between the 2 groups. There was a statistically significant difference in the lung SUV_max_ (*P* < .001) and the SUR-BP (*P* = .001) after CHT between the 2 groups.

Finally, patient diagnoses were based on their clinical diagnoses and treatment, and their clinical symptoms and any adverse imaging findings disappeared following CHT or (steroid or anti-inflammatory) treatment.

## 4 Discussion

DILD is a critical side effect in HL and NHL patients treated with CHT, with an incidence rate of 5.5% to 10%.^[4]^ DILD can be expressed as diffuse alveolar damage, acute or chronic alveolar hemorrhage, nonspecific interstitial pneumonia (idiopathic pulmonary fibrosis), hypersensitivity pneumonitis, organizing pneumonia (bronchiolitis obliterans organizing pneumonia), and eosinophilic pneumonia.^[12,13]^ In this study, 50 cases of DILD occurred in 677 lymphoma patients, with an incidence of 7.4%. There are many potential mechanisms of DILD,^[14–17]^ including indirect cytotoxic effects of the CHT drugs, and damage to the alveolar and pulmonary capillary endothelium cells. In addition, some CHT drugs are transformed into toxic metabolites after entering the human body, or electrophilic conjugates and oxygen free radicals after liver biotransformation, causing damage to cell membrane structures and functions. Also, lymphocytes and macrophages release various inflammatory factors (such as tumor necrosis factor and interleukins) that activate neutrophils, fibroblasts, and alveolar macrophages, causing lung injury or fibrosis. Moreover, CHT drugs lead to the release of cytokines, and changes in capillary permeability, leading to pulmonary edema. Finally, a few patients are more susceptible to allergies induced by the CHT drugs, leading to allergic reactions.

DILD is generally clinically asymptomatic, with a few patients suffering from a cough and fever. The main CHT regimens involved include R-CHOP, R-CDOP, and ABVD. This study included 553 patients with NHL and the following treatments: 277 R-CHOP, 97 CHOP, 38 R-CDOP, 17 CDOP, and 124 others. There were also 124 patients with HL and they had the following treatments: 109 ABVD and 15 others. DILD patients who received R-CHOP (29 cases) underwent the most CHT regimens, accounting for 10.5% of all those undergoing R-CHOP, which is consistent with Zhou research.^[16]^ Generally, R-CHOP is mainly used to treat diffuse large B-cell lymphoma; however, it is difficult to distinguish a single DILD from a comprehensive DILD. CHOP (2 cases) treatment accounted for 2.1% of those with DILD. The R-CHOP regimen showed the highest prevalence of DILD and was more likely to cause DILD than CHOP. Rituximab^[18]^ is a monoclonal antibody that targets the human-mouse chimeric CD20 antigen on the surface of B cells and mediates tumor cell apoptosis by inducing antibody-dependent and complement-dependent cytotoxicity, and it is mainly used for the treatment of NHL.

Studies have shown that the incidence of DILD has gradually increased with the widespread use of CHT drugs,^[19,20]^ and this may be related to the decline in patient immunity as CHT drugs attack normal B lymphocytes and activate cytotoxic T lymphocytes, leading to the release of cytokines and complement activation. The main clinical manifestations include fever, cough, and dyspnea 2 to 3 weeks after treatment, and the imaging shows pulmonary interstitial disease. In addition, R-CHOP elevates the incidence of IP, possibly because of an increased risk for pneumocystis jirovecii pneumonia and fungal infection and this indicates that laboratory tests must be combined to identify both DILD and PI. Cyclophosphamide can also cause DILD. The pathological manifestations are diffuse alveolar injury,^[12]^ which can be relieved following drug withdrawal or glucocorticoid treatment. Doxorubicin can lead to cardiotoxicity or transient neutropenia in a few patients, and lung injury is rare.^[16]^ Vincristine can cause neurotoxicity in a small number of patients.^[21]^

In our study, 7 patients had DILD following R-CDOP treatment. R-CDOP treatment replaces doxorubicin with doxorubicin liposomes. Zhou et al^[21]^ found that R-CDOP causes a higher incidence of DILD than CDOP and R-CHOP (21.1% vs 17.4% and 1.8%). Other clinical studies^[22,23]^ have found that R-CDOP reduces the cardiotoxicity of doxorubicin and increases the incidence of DILD. In addition, a small number of patients may have gastrointestinal reactions and neutropenia.^[24]^ In our study, 5 patients with HL developed DILD after treatment with ABVD. ABVD is commonly used for the treatment of HL. Bleomycin may result in lung inflammation that can progress to hypersensitivity pneumonitis and bronchiolitis obliterans organizing pneumonia, and even fibrosis, with an incidence of 3% to 20%.^[25]^ The underlying mechanism is not clear but may be related to oxidative damage, and a few patients may develop gastrointestinal reactions, lung damage, and bone marrow suppression. In this study, the median CHT cycle of patients with DILD was 4 weeks, which mostly occurred in the middle and late stages of their CHT regimen, consistent with previous studies.^[16,21]^ Therefore, it is necessary to closely monitor patients for possible adverse reactions in the middle and late stages of CHT, and adjust the CHT regimen in time to avoid unnecessary adverse reactions and diseases that can affect a patient's prognosis.

CT imaging allows a precise assessment of the characterization of lesion morphology and distribution,^[15,26]^ and HRCT is the gold standard for evaluating both alveolar and interstitial findings. CT imaging in patients with DILD mostly shows decreased lung light transmittance, diffuse or multi-focal patchy shadows, ground-glass opacities, intralobular septal thickening, and lung consolidation, which is common in the lung periphery and under the pleura.^[10,27]^ More seriously, there can be consolidation shadows or pleural effusions, and a few patients develop allergic pneumonia, which can be characterized by a thickened and disordered texture of the lungs with a grid-like mosaic appearance. In our study, FDG uptake was detected at an extremely early stage when no symptoms or abnormal findings were apparent via HRCT.

During treatment, DILD must be differentiated from PI, lymphoma pulmonary infiltration, and pulmonary fibrosis. Some patients may succumb to lung infections due to decreased immunity, which is difficult to distinguish from DILD; however, DILD can generally be identified by laboratory tests (such as routine blood and sputum cultures). In our study, PET/CT showed a normal lung background, scattered or partial patchy shadows, and frosted glass, with high uptake, which can be significantly improved following anti-inflammatory treatment.^[6]^ The laboratory and imaging results of patients before the enrollment were ideal, and other lung diseases and susceptible factors were excluded, to enable a clear distinction between DILD and PI.

Some lymphoma patients experienced pulmonary infiltration at an early stage or during treatment, which manifested as nodules or consolidation in the lungs with a similar radioactive uptake to the primary lesion and was often accompanied by parallel or progressive manifestations of the primary lesion. These were identified based on clinical symptoms and imaging results before and after treatment. Pulmonary fibrosis shows scattered shadows in the lungs, generally without radioactive uptake.

This study had several limitations. The patients were enrolled from the same hospital, and the sample source was homogenous. In addition, some patients were re-examined after CHT, and the examination interval time is too long, DILD patients recovered by themselves during the treatment process, were not found in time. Furthermore, some patients were not enrolled due to imperfect clinical and imaging data, which may have led to bias in the study results.

In summary, ^18^F-FDG PET/CT has a high sensitivity and detection rate for the primary and metastatic lesions of lymphoma. It can be used to detect specific manifestations of DILD after CHT, with clearly identifiable histologic patterns and appearance. This can aid our evaluation of treatment responses and provide guidance for subsequent treatment plans for patients.^[8]^

## Acknowledgments

We greatly thank the equipment support of Shandong Cancer Hospital and Institute, ethical approval by the medical ethics committee, and other teachers’ help. And we appreciate Textcheck for English language editing.

## Author contributions

**Investigation:** Tingting Lu.

**Project administration:** Tingting Lu.

**Writing – original draft:** Tingting Lu.

**Writing – review & editing:** Guoren Yang.
